# Fish DNA Sensors for Authenticity Assessment—Application to Sardine Species Identification

**DOI:** 10.3390/molecules29030677

**Published:** 2024-02-01

**Authors:** Myrto Kakarelidou, Panagiotis Christopoulos, Alexis Conides, Despina P. Kalogianni, Theodore K. Christopoulos

**Affiliations:** 1 Analytical/Bioanalytical Chemistry & Nanotechnology Group, Department of Chemistry, University of Patras, Rio, 26504 Patras, Greece; myrtokakarelidou@gmail.com (M.K.); pkchristop@gmail.com (P.C.); 2 Hellenic Centre for Marine Research, Institute for Marine Biological Resources, 46.7 km Athens-Sounion, Anavyssos, 19013 Attika, Greece; conides@hcmr.gr; 3 Institute of Chemical Engineering Sciences, Foundation for Research and Technology Hellas (FORTH/ICE-HT), Platani, 26504 Patras, Greece

**Keywords:** authentication, adulteration, mislabeling, traceability, *Sardina pilchardus*, *Sardinella aurita*, gold nanoparticles, rapid test

## Abstract

Food and fish adulteration is a major public concern worldwide. Apart from economic fraud, health issues are in the forefront mainly due to severe allergies. Sardines are one of the most vulnerable-to-adulteration fish species due to their high nutritional value. Adulteration comprises the substitution of one fish species with similar species of lower nutritional value and lower cost. The detection of adulteration, especially in processed fish products, is very challenging because the morphological characteristics of the tissues change, making identification by the naked eye very difficult. Therefore, new analytical methods and (bio)sensors that provide fast analysis with high specificity, especially between closely related fish species, are in high demand. DNA-based methods are considered as important analytical tools for food adulteration detection. In this context, we report the first DNA sensors for sardine species identification. The sensing principle involves species recognition, via short hybridization of PCR-amplified sequences with specific probes, capture in the test zone of the sensor, and detection by the naked eye using gold nanoparticles as reporters; thus, avoiding the need for expensive instruments. As low as 5% adulteration of *Sardina pilchardus* with *Sardinella aurita* was detected with high reproducibility in the processed mixtures simulating canned fish products.

## 1. Introduction

Food fraud is a major problem worldwide. Because food fraud is linked to economic interests and health concerns, it is a high priority for food safety and quality worldwide. In addition, consumer demand for correct food labeling is constantly increasing. Along with other animal products, fishery products are considered to be one of the most adulterated foods [1,2]. In addition, fish are among the most easily adulterated foods due to morphological changes during processing that are not visible to the naked eye [3]. An increase in fish adulteration has been observed in recent years, being difficult to control due to the evolution of fraudulent practices. Adulteration is the substitution of one species of fish with another species of lower price and lower quality. Species swaps comprise the most prevalent fish fraud [4]. Sardines are vulnerable to adulteration as they contain valuable nutrients and are widely consumed worldwide. The only species that can be listed as sardine in canned food is the *Sardina pilchardus*, while if other similar species are used such as *Sardinella aurita*, the trade name of “X sardines”, where X is different from *S. pilchardus* species, must be used [5,6].

To control adulteration, various analytical methods have been developed, including spectroscopy, chromatography, and protein-based methods. However, these methods are typically time-consuming, require highly trained personnel, and often use expensive and complex instrumentation and chemometrics for data interpretation [1,3,7]. The latest advances in spectroscopic techniques for authentication of animal-origin food include terahertz spectroscopy, laser-induced breakdown spectroscopy, hyperspectral imaging, nuclear magnetic resonance spectroscopy (NMR), Raman spectroscopy, near-infrared- and mid-infrared spectroscopy, Fourier transform infrared (FT-IR) spectroscopy. UV–Vis and fluorescence spectroscopy have the advantage of being non-destructive, but provide often low distinction ability between closely-related species [4,8,9,10]. DNA-based methods are preferred for fish identification, especially for canned fish, because DNA is characteristic of each species and resistant to food processing conditions, such as the heat treatment during canning. Therefore, DNA-based methods are considered valuable analytical tools against food fraud [3]. In addition, among the molecular methods, DNA barcoding has gained increased interest, being a successful tool to correctly identify animal species. Target gene selection is crucial to be able to discriminate between closely related species [7]. Mitochondrial DNA is widely used for species identification as it has a high number of gene copies and is ideal especially for highly processed food products or for a small amount of tissue sample. Finally, next-generation sequencing (NGS) has come to the front to overcome the limitations of DNA-barcoding [11].

The molecular methods for identifying sardine species include polymerase chain reaction followed by restriction fragment length polymorphism analysis (PCR-RFLP) [12,13,14], DNA sequencing [6,15,16], PCR followed by agarose gel electrophoresis [17,18,19], exon-primed intron-crossing (EPIC) PCR with acrylamide gel electrophoresis [20], real-time PCR with SYBR Green as fluorescent dye to detect the amplicons [21], real-time PCR using double-labelled detection probes with a fluorescent molecule and a quencher [5,22], and real-time PCR followed by melting curve analysis or high-resolution melting curve analysis [23,24]. The above methods either use specialized and expensive instrumentation or are time-consuming and have a low throughput.

In the present work, we have developed fish DNA sensors in a simple rapid-test format, that enable visual identification of the two main sardine species, *Sardina pilchardus* and *Sardinella aurita*. The sensing principle includes species recognition by hybridization of PCR-amplified sequences with specific probes, capture in the test zone of the sensor, and detection by the naked eye using gold nanoparticles as reporters, eliminating the need for expensive instruments. In contrast to other methods, the proposed method provides shorter analysis time, and allows the detection of as low as 1% adulteration in mixtures of PCR products and 5% adulteration in tissue mixtures of processed (canned) samples of both species. To our knowledge, this is the first report of fish DNA sensors for the identification of sardine species.

## 2. Results

In this study, a molecular rapid test was developed for the detection of fish adulteration for the species *S. pilchardus* with *S. aurita*. For this purpose, DNA was first isolated from the fresh tissue of both fish species, as well as from processed mixtures of the two species. The DNA was subjected to amplification by PCR using a common primer pair for the two species. The PCR products were 181 bp and 204 bp for *S. pilchardus* and *S. aurita*, respectively. The products were analyzed by electrophoresis in a 2% agarose gel using 2 μL of each PCR product. Quantification of the products was performed using the free-online ImageJ-gel Analyzer software (National Institutes of Health and the Laboratory for Optical and Computational Instrumentation, LOCI, University of Wisconsin, WI, USA). The DNA fragments were compared with the commercial ΦX174 DNA-HaeIII DNA marker (500 ng) (Figure S1).

**Figure 1:**
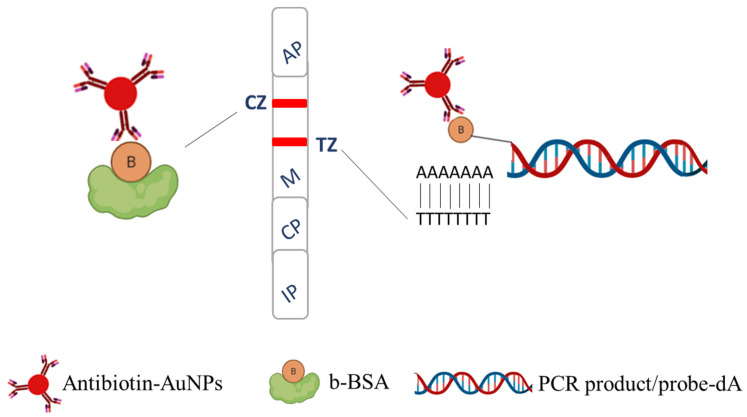
Principle of the fish DNA sensor. B: biotin, AuNPs: gold nanoparticles, IP: immersion pad, CP: conjugate pad, M: membrane, AP: absorbent pad, A: adenine, T: thymine, CZ: control zone, and TZ: test zone.

The identification of the two sardine species was performed by using two different species-specific DNA probes that were hybridized to the amplified sequences followed by detection with the fish DNA sensor. The principle of the method is illustrated in Figure 1. The PCR products were biotinylated and the specific detection probes carried a poly-dA tail at one end. The hybrids were captured from the immobilized poly-dT sequences at the test zone of the sensor and detected by antibiotin-AuNPs through interaction of the anti-biotin Ab with the biotin moiety of the amplification product, forming a red line. A second red line was visualized at the control zone of the strip, as the excess of antibiotin-AuNPs was accumulated to the immobilized biotinylated BSA.

### 2.1. Effect of the Treatment of the Sample to the Optical Signal of the Test Zone of the Strip

**Figure 2:**
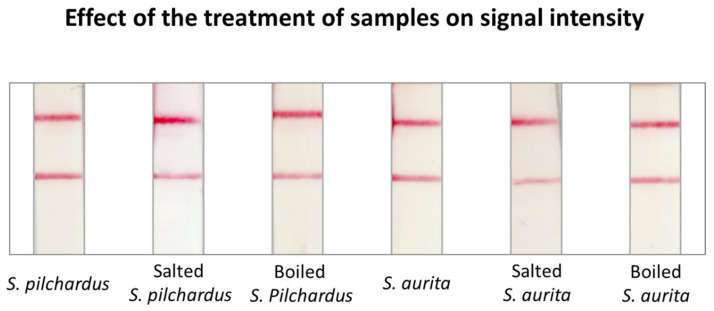
Effect of the treatment of fish samples to the signal of the test zone of the DNA sensor. Samples were treated with salt and boiling in the presence of various ingredients to simulate canned conditions.

We initially investigated whether the type of sample (processed and unprocessed) influenced the intensity of the color of the test zone of the sensor. For this purpose, PCR products from both fresh and cooked/processed samples were hybridized at a concentration of 20 nM with the species-specific probes. The fresh samples of both species were found to have only a slightly stronger signal than the processed samples, making the proposed method suitable for detecting adulterations in processed fish samples (Figure 2).

### 2.2. Effect of the Amount of the Amplification Product on the Signal

**Figure 3:**
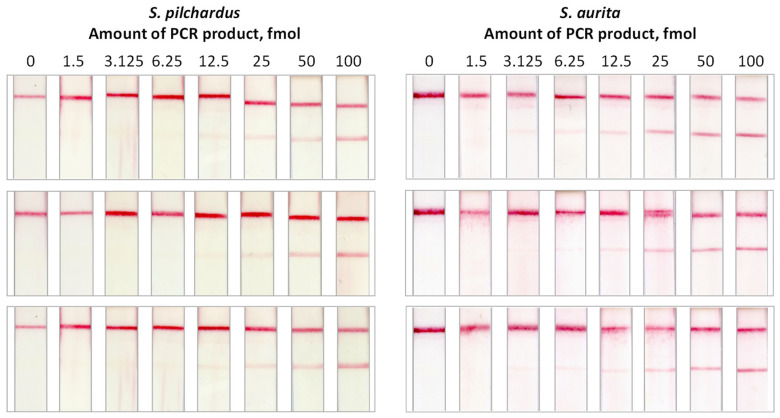
Effect of the amount of the amplification product on the signal of the DNA sensor. Various amounts of PCR product, from *S. pilchardus and S. aurita*, ranging from 0 to 100 fmol were analyzed in triplicate with the DNA sensor.

The effect of the amount of PCR product on the signal at the test zone of the strip was studied as follows: The amplification products from *S. pilchardus* and *S. aurita* were separated by electrophoresis and quantified by imaging of the ethidium bromide-stained agarose gels. Serial two-fold dilutions of the products were then prepared and 5-μL aliquots containing 100, 50, 25, 12.5, 6.25, 3.125, and 1.56 fmol DNA were used for hybridization with the species-specific probes and application to the DNA sensor. Negative samples that contained no target DNA were also included in the study. All samples were analyzed in triplicates. It was observed that the intensity of the color band at the test zone of the sensor increased with increasing amount of the PCR product (fmol) applied to the sensor. As low as 6.25 fmol of PCR product from *S. pilchardus* and 3.13 fmol of the PCR product from *S. aurita* were detectable by the proposed DNA sensor (Figure 3).

### 2.3. Cross-Hybridization Study

**Figure 4:**
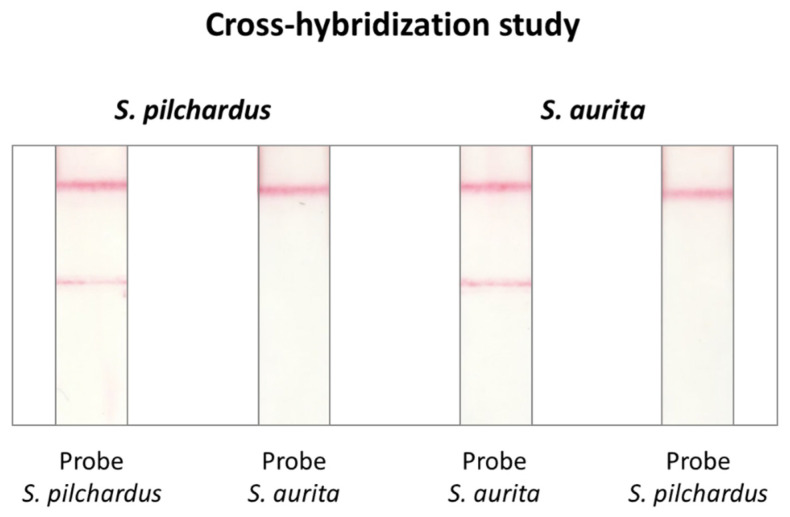
Cross-hybridization study for the experimental confirmation of probe specificity. Amplification products from *S. pilchardus* and *S. aurita* were hybridized, separately, to both probes and analyzed with the DNA sensor.

In this study, the specificity of the probes was examined, i.e., whether *S. pilchardus* probe hybridized with *S. aurita* DNA and whether the *S. aurita* probe hybridized with *S. pilchardus* DNA. PCR products from *S. pilchardus* and *S. aurita* were hybridized, separately, with both available probes. A-1.5 μL volume (150 fmol) of each PCR product was hybridized to 0.5 pmol of the *S. pilchardus* probe and *S. aurita* probe and the hybrids were detected by the DNA sensor. The results are shown in Figure 4. It was observed that a red line was obtained in the test zone of the sensor only when the PCR product hybridized with its complementary species-specific probe; otherwise, no signal was visible.

### 2.4. Method Performance Evaluation Using Mixtures of Amplification Products

**Figure 5:**
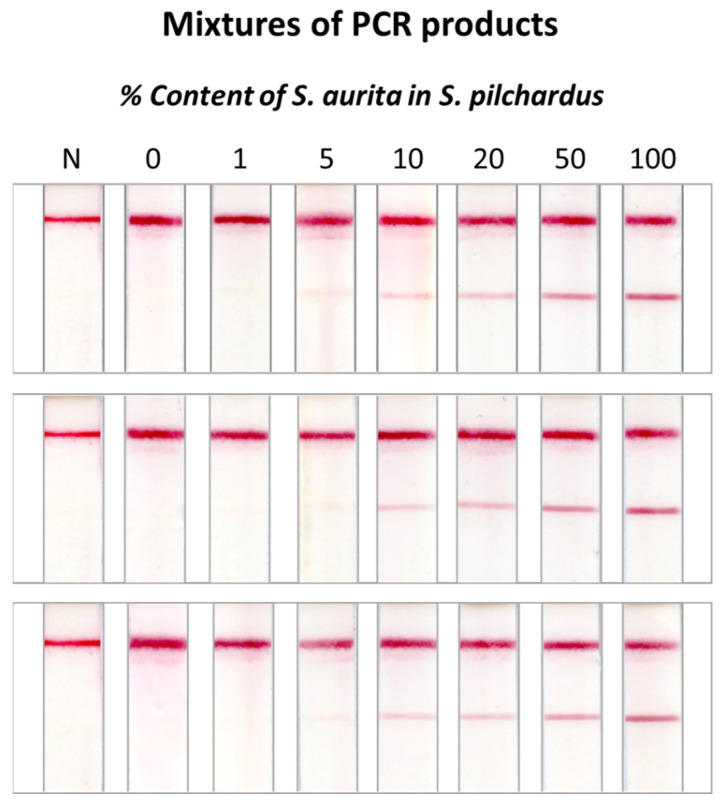
Method performance evaluation using mixtures of amplification products from *S. pilchardus* and *S. aurita*. The samples were analyzed in triplicate with the DNA sensor.

Mixtures of the PCR products were prepared, containing different percentages of the *S. aurita* PCR product (0, 1, 5, 10, 20, 50, and 100%) in the *S. pilchardus* PCR product. The study was conducted in triplicate. The mixtures were hybridized with the specific probe for *S. aurita* and an amount of 100 fmol of the hybrids was applied to the DNA sensor. The results are shown in Figure 5. It was observed that adulteration of *S. pilchardus* with *S. aurita* was detected up to 1%. The mixtures were also tested by hybridization with the *S. pilchardus*-specific probe as a positive control of the entire procedure (see Figure S2). As the percentage of adulteration increased, a slight change in signal intensity was observed, while at a level of adulteration of 100%, no signal was obtained in the test zone of the DNA sensor due to the absence of *S. pilchardus* DNA.

### 2.5. Method Performance Evaluation Using Mixtures of Processed Samples

**Figure 6:**
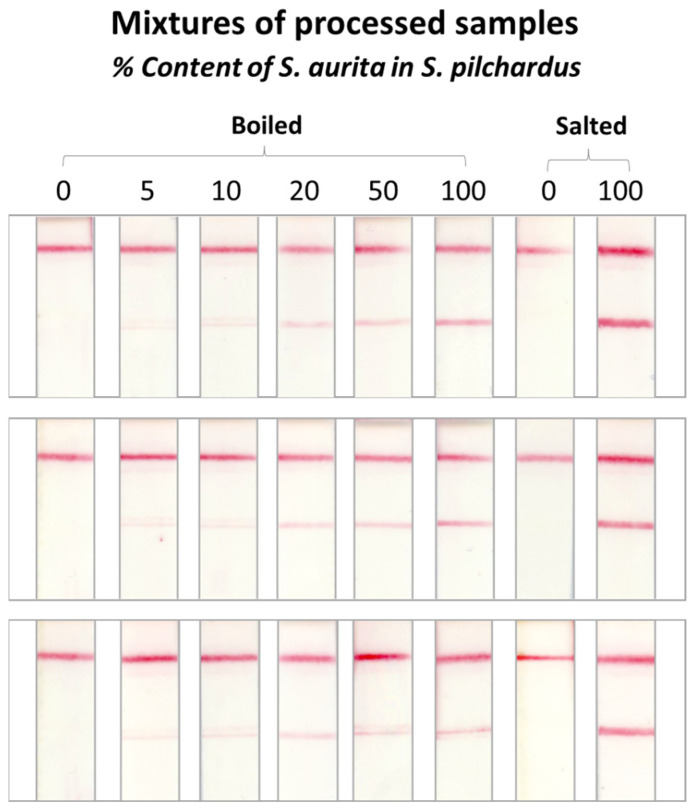
Method performance evaluation using mixtures of processed (canned) samples. The samples were analyzed in triplicate with the DNA sensor.

Mixtures of the two fish species were prepared by mixing tissues of both species with oil and paprika at different adulteration ratios (0–100%). The mixtures were cooked to simulate canned conditions. DNA was then isolated from the processed samples. The isolated DNA was subjected to PCR and the products were analyzed in triplicate with the proposed DNA sensor. Salted *S. pilchardus* (0% adulteration) and *S. aurita* (100% adulteration) were also analyzed as above. The PCR products of the boiled samples were diluted three times with 1× TE buffer, pH 8.0, while the salted *S. pilchardus* was diluted five times. Hybridization with the species-specific probes was performed, using a 1-μL volume of the diluted PCR product. The DNA sensor results are shown in Figure 6 for the *S. aurita*-specific probe (*n* = 3) and in Figure S3 for the *S. pilchardus*-specific probe. For the processed mixtures (cooked), the adulteration level detected was 5% with high repeatability.

### 2.6. Repeatability of the DNA Sensor

**Figure 7:**
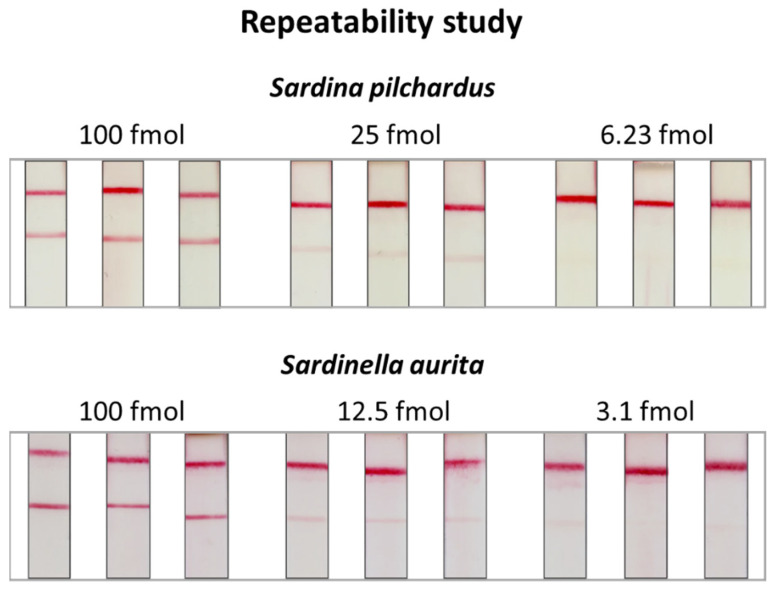
Repeatability of the DNA sensor. Different amounts of PCR products of *S. pilchardus* and *S. aurita* were analyzed in triplicate with the DNA sensor.

Finally, the repeatability of the DNA sensor was assessed. Different amounts of PCR products of both fish species were analyzed in triplicate with the DNA sensor. The results are presented in Figure 7. The images of the strips were obtained using a regular benchtop scanner. After densitometric analysis of the test zones of the strips, the coefficient of variations, $\%CV=\frac{SD}{\bar{x}}\times 100$, were calculated and found to be 3.6, 4.4, and 7.8% for the 6.3, 25, and 100 fmol of *S. pilchardus* and 17.4, 9.6, and 8.1% for the 3.1, 12.5, and 100 fmol of the PCR product of *S. aurita*, proving a very good repeatability of the proposed DNA sensor.

## 3. Discussion

Fish authentication is a major concern worldwide. For these reasons, new methods for fish species identification are of great importance. DNA-based methods are the preferred ones due to DNA stability and enhanced sensitivity. Herein, we have developed a DNA sensor for visual discrimination of *S. pilchardus* from each most common adulterant *S. aurita*. 

So far, few methods have been reported for authenticity testing of sardines. More specifically, in 2016, PCR-RFLP and DNA sequencing for confirmation were used and compared by Leonardo et al. for the discrimination of several sardine species in canned products. The cytochrome b mitochondrial gene was used in this study, as well as phylogenetic analysis which was also conducted in the samples. As a conclusion, RFLP was not adequate to allow discrimination of the species and had a low throughput. On the other hand, DNA sequencing allowed unambiguous identification of different sardine species [13]. Also, in 2012 and 2003, a PCR-RLFP method combined with phylogenetic analysis was developed. A fragment of the cytochrome b gene of the mitochondrial DNA was exploited to discriminate *S. pilchardus* from other species in both fresh and canned products [12,14]. Lago et al., in 2011, used PCR, DNA sequencing, and phylogenetic analysis for the identification of different single nucleotide polymorphisms (SNPs) in the cytochrome b mitochondrial gene of *S. pilchardus* and *S. aurita*. This method could be applied to all kinds of canned food, regardless of the treatment that fish samples have undergone. The method was also applied to mixtures of tissues of both species, and down to 5% (*w*/*w*) of the tissue of *S. aurita* was detected [6]. Direct sequencing methods were also applied for species identification in fresh and canned products based again on mitochondrial DNA cytochrome b or the cytochrome oxidase I gene. The results were assessed by phylogenetic analysis [15,16]. PCR based on the 16S RNA mitochondrial gene, followed by agarose gel electrophoresis was used for identification of *S. pilchardus* [17,19] and *S. pilchardus*, *S. aurita*, and *S. maderensis* [18]. Exon-primed intron-crossing (EPIC) PCR with acrylamide gel electrophoresis was also exploited for *S. pilchardus* identification [20]. DNA barcoding and real-time PCR based on SYBR Green for detection of amplicons was used for *S. pilchardus* screening in commercial fish products [21]. Taqman probes with real-time PCR was introduced to increase the specificity of the method and applied for the discrimination of *S. pilchardus* from *S. aurita* in commercial products. An amount of 25 pg was adequate for positive results. Processed products had higher Cq values than fresh samples, while Cq remained <30 for all samples [5]. The same method was developed for the discrimination of *S. pilchardus* from anchovy with a detection limit of 0.05 ng of DNA [22]. Finally, real-time PCR with subsequent (high-resolution) melting curve analysis for confirmation of the results was developed for *S. pilchardus* identification [23,24]. All the above methods usually require expensive instrumentation or are highly time-consuming. PCR-RLFP, however, has a lower distinction capability and cannot provide quantitative results. The present work reports the first DNA sensor developed for sardine authenticity testing. It enables rapid and visual discrimination by the naked eye of down to 5% of adulteration of *S. pilchardus* with *S. aurita* with high discrimination capability. However, it can only provide semi-quantitative results. The sensor is easy to develop and use, without the need for special and expensive instrumentation. Therefore, it can be easily adopted by any laboratory and public authorities for authenticity testing. Also, one of the great advantages of the proposed DNA sensor is its universality and versatility. The sensor can be used for the identification of any other fish species as the molecular recognition by species-specific primers and probes is performed prior to the application to the sensor. Finally, the proposed DNA sensor can be modified accordingly to be able to detect more than two different species-specific DNA sequences on the same sensor. A comparative Table S1 is available in the Supplementary Material.

## 4. Materials and Methods

### 4.1. Materials

Oligonucleotides, primers, and probes were purchased from Eurofins Genomic (Vienna, Austria). The Nucleospin Tissue kit for DNA extraction was obtained from Macherey-Nagel (Duren, Germany). Kapa 2G Fast polymerase with Buffer A was from Kapa Biosystems (Basel, Switzerland), dNTPs and dUTP from Invitrogen (CarlsBad, CA, USA), FX174 DNA-HaeIII DNA marker and the terminal transferase kit containing the enzyme, the reaction buffer, and the CoCl_2_ from New England Biolabs (Ipswich, MA, USA), and agarose from Fisher BioReagents (Waltham, MA, USA). Bovine serum albumin (BSA) was purchased from Serva Electrophoresis (Heidelberg, Germany), NaHCO_3_ from Fisher Scientific (Loughborough, UK), and EZ-Link Sulfo-NHS-LC-LC-Biotin from Thermo Fisher Scientific (Waltham, MA, USA). The 40 nm gold nanoparticles (0.15 nM) were from BBI solutions (Crumlin, UK) and the anti-biotin antibody from Sigma-Aldrich (Saint Louis, MO, USA). EDTA, borax, SDS, and sodium azide were all obtained from Merck (Darmstadt, Germany). For the construction of the strips, Immunopore FP membrane and glass fiber conjugate pad were purchased from GE Healthcare Life Sciences (Buckinghamshire, UK), whereas the absorbent pads were from Schleicher & Schuell (Dassel, Germany). Finally, for the running buffer, glycerol was obtained from Carlo Erba (Barcelona, Spain), the phosphate salts from Lachner (Neratovice, Czech Republic), and Tween-20 from Fluka (Sigma-Aldrich). The 1× phosphate-buffered saline solution (PBS) pH 7.4 consisted of 137 mM NaCl, 2.7 mM KCl, 8 mM Na_2_HPO_4_, and 2 mM KH_2_PO_4_ and the 6× saline sodium citrate buffer (SSC) pH 7.0 consisted of 900 mM sodium chloride and 90 mM sodium citrate. 

MJ Research PTC-150 Minicycler was used for PCR amplification. The TLC CAMAG Linomat 5 (Muttenz, Switzerland) and UVP Crosslinker CL-3000 by Analytik Jena (Upland, CA, USA) were used for the deposition and the immobilization of the reagents on the membrane, respectively. Finally, a common scanner (EPSON Perfection V600 PHOTO, Seiko Epson Corporation, Suwa, Japan) was used for acquiring the images of the strips.

### 4.2. Samples

Fresh fish samples of *S. pilchardus* and *S. aurita* were collected from local fish monger markets in Western Greece. The samples were kept at −20 °C until analysis.

### 4.3. Sample Preparation

Fresh samples were used unprocessed for method development, while processed samples were also prepared to simulate the types of processed fish-based products available in the market. Two fresh samples of each species were processed in two different ways. One sample was boiled with oil and paprika, to simulate canning conditions, and the other sample was left with salt, oil, and vinegar to obtain a salt-preserved product. Mixtures of both species were also prepared using the cooking procedure as described above. The binary mixtures contained different percentages of adulterants (5%, 10%, 20%, 50%, 100%). All samples were stored at −20 °C.

### 4.4. DNA Extraction

DNA was isolated from fresh samples of *S. pilchardus* and *S. aurita* and from processed mixtures of the two species. Part of the fish dorsal muscle tissue from each sample was cut for DNA isolation. An amount of 25 mg of tissue was used for fresh samples while 24 mg of tissue was cut and weighed directly from processed samples. For the boiled samples, 25 mg of tissue from both species was used for DNA extraction. The Nucleospin Tissue kit was used for DNA isolation according to the manufacturer’s instructions. Elution at the final stage was performed with 50 μL of elution buffer and the isolated DNA was stored at −20 °C.

### 4.5. Primer Design

Ribosomal RNA sequences for *S. pilchardus* (JN590272.1) and *S. aurita* (JN590273.1) were obtained from the NCBI database and sequence alignment was performed using the online free software Clustal Omega (EMBL’s European Bioinformatics Institute (EMBL-EBI, Cambridgeshire, UK). The primers and probes sequences were then studied in terms of secondary structures using the Oligo Analyzer Tool (Integrated DNA Technologies, Coralville, IA, USA) and BLAST (NIH, Bethesda, MD, USA) to confirm their specificity to each species. The sequences of the PCR primers and the probes used throughout this work are presented in Table 1.

### 4.6. Polymerase Chain Reaction (PCR)

PCR was performed using the same primers for both species. The reaction was performed in a final volume of 20 μL and contained 0.8 μM of biotinylated forward primer (Sard Forward), 0.8 μM of reverse primer (Sard Reverse), 1× Kappa 2G buffer A, 0.2 mM each of dATP, dGTP, dCTP, dUTP, 0.25 μL of Kapa 2G Fast polymerase (1.25 U), and 5 μL of isolated DNA from fresh samples or 50 ng of isolated DNA from the mixtures. Amplification was performed as follows: 94 °C for 5 min followed by 35 cycles of 94 °C for 20 s, 52 °C for 20 s, 72 °C for 20 s, and final extension at 72 °C for 7 min. 

### 4.7. Construction of the Sensing Zones of the Device

#### 4.7.1. Biotinylation of Bovine Serum Albumin (b-BSA)

BSA was biotinylated for the construction of the control zone of the sensor. Briefly, 0.2 mg of BSA (200 μL from 1 g/L) was added to 50 μL of 0.5 M NaHCO_3_, pH 9.1, followed by the addition of 1 mg of sulfo-NHS-LC-LC-biotin and incubation for 1 h, at room temperature. 

#### 4.7.2. Insertion of a Poly-dT Tail into the Capture Probe

For the construction of the test zone of the sensor, a poly-dT tail was incorporated into the 3’ end of an oligonucleotide (capture probe) carrying 30 thymine bases (dT_30_). Reaction was carried out in a final volume of 20 μL, containing 1× Reaction TdT Buffer, 0.25 mM CoCl_2_, 5 mM dTTP, 0.05 mM oligonucleotide dT_30_, and 1.5 U terminal transferase (TdT). The solution was incubated at 37 °C for 1 h. To stop the reaction, 2 μL of a 0.5 M EDTA solution, pH 8.0, was added and the solution was stored at −20 °C.

### 4.8. Insertion of a Poly-dA Tail into the Detection Probes

For the detection of PCR products with the DNA sensor, a poly-dA tail was inserted into the species-specific detection probes for *S. pilchardus* and *S. aurita*. The reaction conditions were as above except that 2 mM dATP, 0.02 mM of each probe, and 1 U of TdT were used.

### 4.9. Coupling of Anti-Biotin Antibody to Gold Nanoparticles (Antibiotin-AuNPs)

A-1 mL volume of AuNPs (0.15 nM) was centrifuged at 3300× *g* for 20 min and 500 μL of the supernatant was discarded. The pH was then adjusted to 9.0 with the addition of a proper volume of 200 mM borax. Then, 4.6 μg of anti-biotin antibody, dissolved in 2 mM borax, was added gradually under stirring to a final concentration of 0.01 g/L. The solution was incubated for 45 min in the dark and 100 μL of 100 g/L BSA in 20 mM borax were added for blocking. The solution was incubated for another 10 min at room temperature. This step was followed by centrifugation at 4500× *g* for 15 min, washing with 500 μL of wash solution (10 g/L BSA in 2 mM borax), centrifugation at 4500× *g* for 5 min, and redispersing in 100 μL of redispersion solution (1 g/L BSA and 1 g/L NaN_3_ in 2 mM borax). Antibiotin-AuNPs were finally stored at 4 °C. A 4-μL aliquot of antibiotin-AuNPs was deposited on the conjugate pad of the strip-type DNA sensor.

### 4.10. Assembly of the Fish DNA Sensor

The strip-type DNA sensor (4 mm × 70 mm) consisted of a plastic substrate, on which the individual pieces of absorption pad, nitrocellulose membrane, conjugate pad, and immersion pad were assembled. On the nitrocellulose membrane, b-BSA and poly-dT were immobilized to create the control zone and the test zone, respectively. More specifically, a solution containing 10 μM poly-dT in 5% (*v*/*v*) MeOH, 2% (*w*/*v*) sucrose and 6× SSC pH 7.0 and a solution containing 1.5 or 2.5 mg/L b-BSA in 5% (*v*/*v*) MeOH, 2% (*w*/*v*) sucrose and 1× PBS buffer pH 7.4 were deposited at specific areas on the nitrocellulose membrane, using the commercial dispenser Linomat 5. The membrane was finally dried in a UV crosslinker at 125 mJ/cm^2^ for 15 min. Subsequently, the strip was properly assembled as follows. Double-sided tape was taped onto a plastic substrate and then the nitrocellulose membrane was attached. The absorbent pad was placed on top of the membrane, the conjugate pad was placed in the section below the membrane, and the immersion pad was placed below the conjugate pad, in such a way that there was an overlap between all the pads. Finally, the assembly was cut to 4 mm width per sensor.

### 4.11. Detection of S. pilchardus and S. aurita

Each PCR product was hybridized to the specific probe carrying a poly-dA tail prior to the application to the sensor. A volume of 1.5 μL of the biotinylated PCR product was mixed with 1.5 μL of 0.5 μΜ poly-dA specific probe and 1.5 μL of 0.4 M NaOH. The solution was incubated at room temperature for 5 min and 1.5 μL of 0.4 M Tris-HCl and 1.5 μL of 0.4 M HCl were then added. The mixture was finally incubated at 42 °C for 10 min. Subsequently, a 5-μL volume was applied to the conjugate pad and the strip was immersed into 400 μL of the running buffer (1× PBS, 1% (*w*/*v*) BSA, 1% (*v*/*v*) Tween-20, 1% (*v*/*v*) glycerol, 0.35% (*w*/*v*) SDS). The strip was removed from the running solution after 15 min and scanned by a regular scanner.

## 5. Conclusions

We have developed a fish DNA sensor for fish authentication in a rapid-test format. The method involves detecting adulteration of *Sardina pilchardus* with *Sardinella aurita* using species-specific DNA probes. The developed DNA sensor is the first DNA sensor reported for the identification of sardine species and particularly for the detection of adulteration of *S. pilchardus*. Detection is performed visually using gold nanoparticles as reporters. Compared to existing DNA-based fish adulteration methods, the method is simple and rapid. The sensor is cost-effective (<2 €), easy to use, and very practical as it can be developed in the form of a dry-reagent strip-type sensor; no qualified personnel or costly instrumentation is required, while it can be used by authorities for fish authentication control or any laboratory including fish processing industries. Finally, the method was successfully applied to fresh and processed samples treated by cooking and salting in the presence of other ingredients to simulate canning conditions. For mixtures of the two species, as low as 5% adulteration was detected in the processed samples with very good repeatability.

## Figures and Tables

**Table 1:** Sequences of primers and probes for *S. pilchardus* and *S. aurita* [4].

	Name	Sequence (5′→3′)
**Primers**	Sard Forward	Biotin-CGGTGGTMAAACACATG
	Sard Reverse	GTCTGATCTGAGGTCGT
**Probes**	*S. pilchardus* Probe	CCTTGCTCCAGAGGTCCG
	*S. aurita* Probe	CCTYGCTCTACGGTCCGG

## Data Availability

Data are available upon requirement.

## Supplementary Materials

The following supporting information can be downloaded at: https://www.mdpi.com/article/10.3390/molecules29030677/s1, Figure S1: Electropherogram of PCR products for *S. pilchardus* with *S. aurita*.; Figure S2: Mixtures of PCR products. The mixtures contained 0–100% PCR product from *S. aurita* in PCR product from *S. pilchardus* with a total amount of 100 fmol on the strip; Figure S3: Mixtures of processed samples. The mixtures contained 0–100% of *S. aurita* (tissue) in *S. pilchardus*; Table S1: Comparison of methods for sardines’ adulteration detection.
