# Supplementary material for: Fish DNA Sensors for Authenticity Assessment—Application to Sardine Species Identification
Source: Molecules. 2024 Feb 1;29(3):677. doi: 10.3390/molecules29030677 (PMC10856316; doi:10.3390/molecules29030677)
Supplement: Supplementary file 1 [file molecules-29-00677-s001.zip › molecules-2824519-supplementary.pdf]

Supplementary material

Fish DNA Sensors for Authenticity Assessment—Application to Sardine Species Identification

Myrto Kakarelidou [1], Panagiotis Christopoulos [1], Alexis Conides [2], Despina P. Kalogianni [1,*] and Theodore K. Christopoulos [1,3,*]

[1] Analytical/Bioanalytical Chemistry & Nanotechnology Group, Department of Chemistry, University of Patras, Rio, 26504 Patras, Greece; myrtokakarelidou@gmail.com (M.K.); pkchristop@gmail.com (P.C.)

[2] Hellenic Centre for Marine Research, Institute for Marine Biological Resources, 46.7 km Athens-Sounion, Anavyssos, 19013 Attika, Greece; conides@hcmr.gr

[3] Institute of Chemical Engineering Sciences, Foundation for Research and Technology Hellas (FORTH/ICE-HT), Platani, 26504 Patras, Greece

* Correspondence: kalogian@upatras.gr (D.P.K.); tchrist@upatras.gr (T.K.C.)

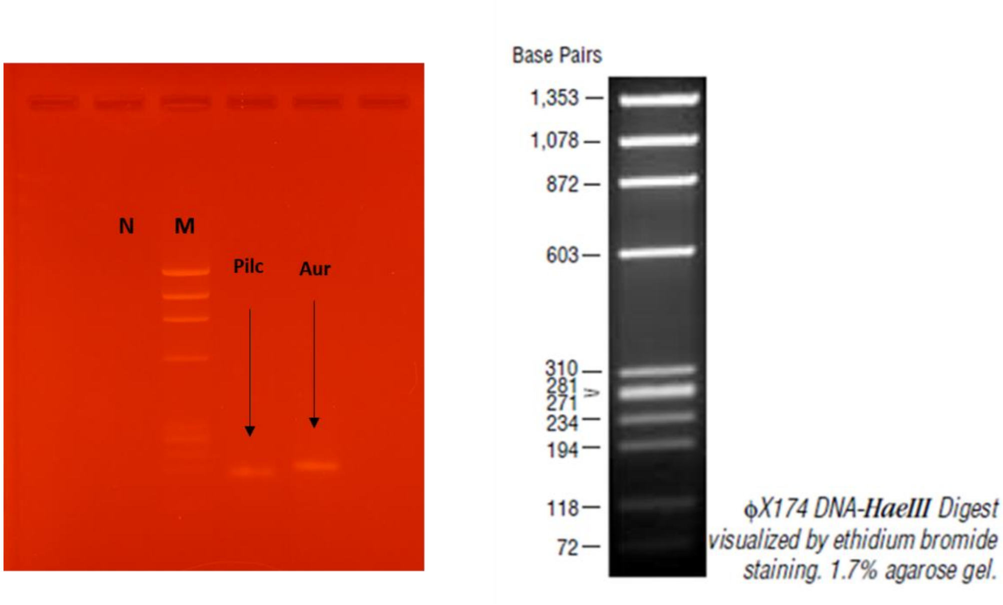

Figure S1. Electropherogram of PCR products for *S. pilchardus* with *S. aurita*. N: negative, M: DNA marker (ΦX174 DNA HaeIII Digest on the right), Pilc: *S. pilchardus* and Aur: *S. aurita*.

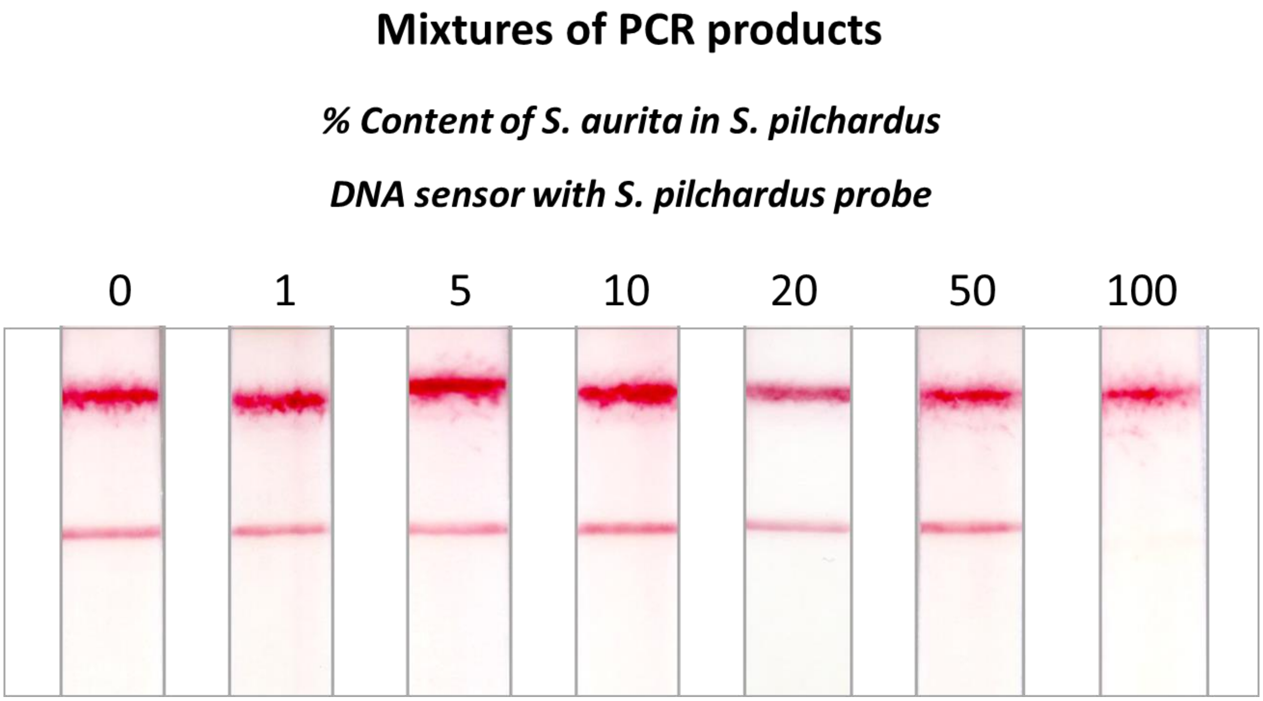

Figure S2. Mixtures of PCR products. The mixtures contained 0 – 100 % PCR product from *S. aurita* in PCR product from *S. pilchardus* with a total amount of 100 fmol on the strip.

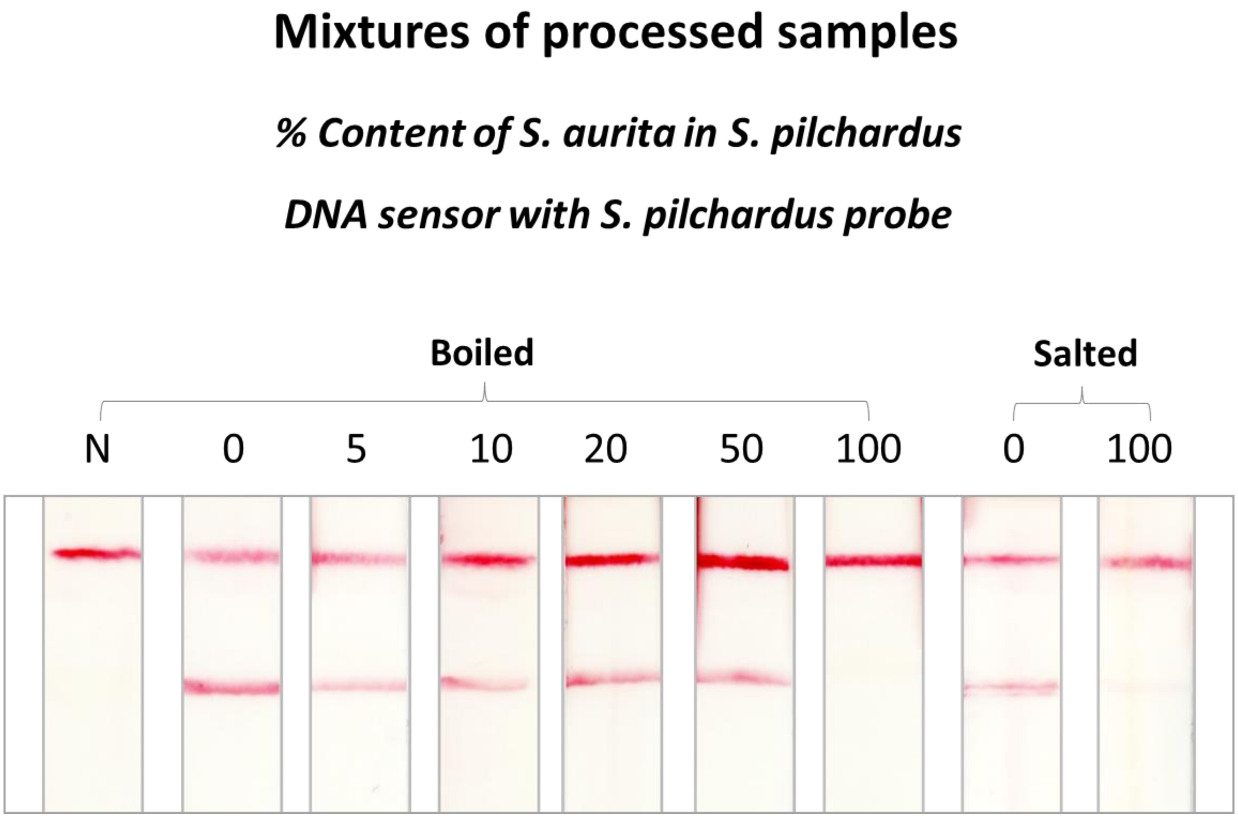

Figure S3. Mixtures of processed samples. The mixtures contained 0 – 100 % of *S. aurita* (tissue) in *S. pilchardus*.

Table S1. Comparison of methods for sardines' adulteration detection

Method	Species examined	LOD	Discrimination capability	Quantitation capability	Ref.
PCR-RFLP and phylogenetic analysis	*S. pilchardus*<br>*S. aurita*<br>*S. melanostictus*<br>*S. caeruleus*<br>*S. maderensis*	-	Low	-	12
PCR-RFLP and DNA sequencing	*S. pilchardus*<br>*S. aurita*<br>*S. brasiliensis*<br>*S. sagax*<br>*S. caeruleus*	-	Low for PCR-RFLP and high for DNA sequencing	✓<br>for DNA sequencing	13
PCR-RFLP and phylogenetic analysis	*S. pilchardus*<br>*S. aurita*	-	Low	-	14
DNA sequencing and phylogenetic analysis	*S. pilchardus*<br>*S. aurita*	5%	High	✓	6
DNA sequencing and phylogenetic analysis	*S. pilchardus*<br>*S. aurita*<br>*S. sagax*<br>*S. caeruleus*<br>*S. melanostictus*<br>*S. maderensis*<br>*S. longiceps*	-	High	✓	15
DNA sequencing and phylogenetic analysis	*S. pilchardus*<br>*S. aurita*<br>*S. longiceps*<br>*S. lemuru*<br>*S. brasiliensis*<br>*S. gibbose*<br>*S. jussieu*<br>*S. fimbriata*<br>*S. tawilis*	-	High	✓	16
PCR and agarose gel electrophoresis	*S. pilchardus*	-	Medium	-	17
PCR and agarose gel electrophoresis	*S. pilchardus*<br>*S. aurita*<br>*S. maderensis*	-	Medium	-	18
PCR and agarose gel electrophoresis	*S. pilchardus*	-	Medium	-	19
Exon-primed intron-crossing (EPIC) PCR with acrylamide gel electrophoresis	*S. pilchardus*	-	Medium	-	20
Real-time PCR / SYBR Greeb	*S. pilchardus*	-	Medium	✓	21
Real-time PCR / Taqman probes	*S. pilchardus*<br>*S. aurita*	-	High	✓	5
Real-time PCR / Taqman probes	*S. pilchardus*	-	High	✓	22
Real-time PCR / Melting curve analysis	*S. pilchardus*	-	High	✓	23
Real-time PCR / High-resolution Melting curve analysis (HRM)	*S. pilchardus*	-	High	✓	24
DNA sensor	*S. pilchardus*<br>*S. aurita*	5%	High	-	This work
